# Aging modulates homeostatic leukocyte trafficking to the peritoneal cavity in a sex-specific manner

**DOI:** 10.1093/jleuko/qiad053

**Published:** 2023-06-13

**Authors:** Sophie J Hopkin, Laleh Pezhman, Jenefa Begum, Dean Kavanagh, Helen M McGettrick, Asif J Iqbal, Myriam Chimen

**Affiliations:** Institute of Cardiovascular Sciences, University of Birmingham, Birmingham, B15 2TT, United Kingdom; Institute of Inflammation and Ageing, University of Birmingham, Birmingham, B15 2TT, United Kingdom; Institute of Cardiovascular Sciences, University of Birmingham, Birmingham, B15 2TT, United Kingdom; Institute of Cardiovascular Sciences, University of Birmingham, Birmingham, B15 2TT, United Kingdom; Institute of Inflammation and Ageing, University of Birmingham, Birmingham, B15 2TT, United Kingdom; Institute of Cardiovascular Sciences, University of Birmingham, Birmingham, B15 2TT, United Kingdom; Institute of Inflammation and Ageing, University of Birmingham, Birmingham, B15 2TT, United Kingdom

**Keywords:** aging, inflammation, leukocyte trafficking, sexual dimorphism, T cells

## Abstract

Aging is associated with exacerbated systemic inflammation (inflammaging) and the progressive loss of immune system function (immunosenescence). Leukocyte migration is necessary for effective immunity; however, dysregulated trafficking of leukocytes into tissue contributes to inflammaging and the development of age-related inflammatory diseases. Aging modulates leukocyte trafficking under inflammatory conditions; however, whether aging modulates leukocyte trafficking under homeostatic conditions remains to be elucidated. Although immune responses are evidently sexually dimorphic, limited studies have investigated the effect of sex on age-related changes to leukocyte trafficking processes. Here, we investigated age-related and sex-specific changes to the leukocyte populations within the peritoneal cavity of young (3-mo), middle-aged (18-mo) and old (21-mo) male and female wild-type mice in the steady state. We found an age-related increase in the number of leukocytes within the peritoneal cavity of female mice, predominantly B cells, which may reflect increased trafficking through this tissue with age. This was accompanied by an increased inflammatory environment within the aged cavity, including increased levels of chemoattractants, including B cell chemoattractants CXCL13 and CCL21, soluble adhesion molecules, and proinflammatory cytokines, which was more pronounced in aged female mice. Intravital microscopy techniques revealed altered vascular structure and increased vascular permeability within the peritoneal membrane of aged female mice, which may support increased leukocyte trafficking to the cavity with age. Together, these data indicate that aging affects homeostatic leukocyte trafficking processes in a sex-specific fashion.

## 1 Introduction

Aging profoundly affects the mammalian immune system, with older mice and humans exhibiting increased circulating levels of proinflammatory mediators^1–3^ and changes to immune cell function^4–8^ when compared with their younger counterparts. Growing evidence suggests that the aging process significantly modulates leukocyte trafficking during inflammation in both humans and mice (reviewed by Hopkin et al.).^9^ For example, immunization of aged (>20 mo) C57Bl6 mice with ovalbumin resulted in reduced CD4^+^ T cell recruitment to the spleen compared with young (<3 mo) mice, which correlated with delayed induction of antigen-specific immunity.^10^ However, much less is known about the impact of age on leukocyte trafficking associated with immunosurveillance and tissue homeostasis.^11–13^ Given this, it is crucial for us to increase our understanding of the impact of age on leukocyte trafficking that leads to weakened immune surveillance in the elderly.

Although it is well established that immune responses are sexually dimorphic in both humans and mice,^14–16^ to date most in vivo studies have used either a single sex or a mixture of male and female participants to dissect the mechanisms underpinning immunological events.^16,17^ Therefore, the impact of biological sex on age-related changes to the immune system has been severely underresearched. There remains controversy over whether such changes are more pronounced in males or females.^18,19^ While older men tend to present with an immune risk profile (i.e., indicative of increased mortality),^20^ older women are more susceptible to developing autoimmune diseases.^16,21,22^ Indeed, during the 2020 SARS-CoV-2 pandemic, older men (63 to 76 yr old) had increased mortality rates following SARS-CoV-2 infection compared with older women.^23^ However, healthy older women (50 to 60 yr old) have increased circulating levels of IL-6 and cholesterol when compared with older men (48 to 58 yr old), suggesting increased systemic inflammation and increased risk of cardiovascular events in otherwise healthy older women.^24^ It remains unclear as to the extent hormones, sex chromosomes, or gender-dependent environmental cues influence the aging immune system,^18^ as well as the extent of their influence on age-related changes to leukocyte trafficking processes.^9,13^ To understand whether aging effects homeostatic leukocyte trafficking in a sex-dependent manner, we quantified leukocyte populations across multiple tissues in young (3 mo), middle-aged (18 mo) and old (21 mo) male and female mice using flow cytometry. The most striking age-related changes to tissue leukocyte composition occurred in the peritoneal cavity, and this age-dependent phenotype was more pronounced in female mice. Aging significantly increased the number of leukocytes within the peritoneal cavity, which was associated with increases in proinflammatory soluble mediators in the peritoneum indicative of age-related changes to homeostatic leukocyte trafficking. Additionally, we observed that the integrity of the peritoneal membrane was compromised with age and may assist leukocyte trafficking into the peritoneal cavity in the steady state. The sex-specific changes to homeostatic leukocyte trafficking that occur with age likely contribute to the dimorphic immune responses observed in older males and females.

## 2 Materials and methods

### 2.1 Murine tissue collection and processing

Experiments were conducted in accordance with UK Home Office regulations and appropriate ethics. Three-month-old (28.36 ± 2 g male; 22.04 ± 1 g female), 18-mo-old (41.26 ± 6 g ale; 30.90 ± 3 g female) and 21-mo-old (32.73 ± 2 g male; 30.08 ± 1 g female) C57Bl/6J wild-type mice were purchased from Charles River Laboratories and were housed at the University of Birmingham animal unit with free access to food and water. Mice were maintained in an specific pathogen-free environment, determined by quarterly health screening of the unit using Federation of European Laboratory Animal Science Associations approved methods. Mice were fed the rodent 5LF2 diet (IPS LabDiet) and were housed in the animal facility for at least 2 mo prior to experimentation. Environmental conditions were 21 ± 2 ˚C, 55% ± 10% relative humidity, and a 12-h light-dark cycle. Mice were culled by cardiac puncture with blood collected into EDTA-coated Eppendorfs. The peritoneal cavity was lavaged with ice cold 5 mM EDTA. Spleen, right hind limb, and inguinal lymph nodes (iLNs) were isolated and stored in phosphate-buffered saline (PBS). Peritoneal lavage fluid (PLF) was centrifuged at 400 *g* for 5 min: supernatant was stored at −80 °C and cells were resuspended in MACS buffer (0.1 mM EDTA, 0.6% bovine serum albumin in PBS; all from Sigma-Aldrich). The spleen and iLNs were crushed through a 40-μM filter. Bone marrow was flushed out of the right hind limb using PBS. Spleen, iLN, bone marrow, and blood samples were all incubated in RBC lysis buffer (155 mM NH_4_Cl, 12 mM NaHCO_3_, 0.1 mM EDTA; all from Sigma-Aldrich) for 10 min, followed by centrifugation at 400 *g* and then resuspension in MACS buffer.

### 2.2 Flow cytometric analysis

All samples were blocked with FcR blocker (Miltenyi Biotec) prior to staining with the following of antibodies for 20 min at 4 °C, after which samples were washed and fixed with 2% formaldehyde: anti-CD3 PECy7 (clone 145-2c11), anti-CD4 eFluor450 (clone GK1.5), anti-CD8 PE-TexasRed (clone 5H10), anti-CD44 FITC (clone IM7), anti-CD25 AF700 (clone PC61.5), anti-KLRG1 APC-eFluor780 (clone 2F1), anti-CD62L PE (clone MEL-14), anti-CD19-APC (clone 1D3), anti-CD45 APC-CY7 (clone 104), F4/80 FITC (clone BM8), anti-CD11c PE-Cy7 (clone N418), anti-gp38 PE (clone 8.1.1) (all from Thermo Fisher Scientific); anti-CD45.2 BV605 (clone 104), anti-CD23 BV421 (clone B3B4), anti-CD93 BV650 (clone AA4.1), anti-CD43 PerCP-Cy5.5 (clone S7), anti-CD21/35 PE (clone 7G6), anti-Siglec F PE-CF594 (clone E50-2440), Ly6G APC (clone 1A8) (all from BD). Compensation controls were generated using cells isolated from the spleen. Immediately prior to analysis CountBright beads (Invitrogen) and Zombie Aqua (BioLegend) were added and samples were acquired using Fortessa-X20. Data were analyzed offline using FlowJo (V-10.2.6; TreeStar) using the flow cytometry gating strategy depicted in Supplementary Fig. 1. Leukocyte populations were quantified using CountBright beads according to the manufacturer's instructions. Data are presented as the total number of each leukocyte population within a given tissue.

### 2.3 Intravital microscopy

Three-month-old and 21-mo-old male and female C57Bl6 mice were anesthetized by intraperitoneal injection of ketamine hydrochloride (100 mg/kg; Pfizer) and medetomidine hydrochloride (10 mg/kg; Pfizer). The trachea and right common carotid artery were cannulated, and the peritoneal membrane was exposed. Prior to imaging, 50 μL 20 kDA dextran-TRITC and 50 μL 70 kDa dextran-FITC (both at 50 mg/mL; both from Sigma-Aldrich) were administered via the carotid artery. The peritoneal membrane was imaged at 60 min post-dextran administration using an Olympus IX81 inverted microscope. After imaging, the mice were sacrificed via cervical dislocation and the PLF was collected as described previously. The concentration of fluorescent dextrans in the PLF was quantified using a BioTek Synergy HT plate reader and the data presented as µg dextran per mL PLF. Images were analyzed offline using Angiogenesis Analyzer plug-in on Fiji Image J (version 2.1.0; Cambridge) to calculate the number of vessels and total vessel length, number of nodes (points of bifurcation), number of mesh structures (vessel circuits), and total mesh area (all expressed per mm^2^).

### 2.4 Cytokine array

The secretome of the PLF was quantified using the Mouse XL Cytokine Array Kit according to the manufacturer's instructions (ARY028; R&D Systems). Membranes were exposed for 1 min and developed using the Compact X4 Automatic X-ray Film Processor (Xograph). The developed membrane was scanned (Supplementary Fig. 2), and the images were analyzed using ImageJ v2.1.0 (National Institutes of Health). Briefly, regions of interest were drawn around each analyte and the intensity value was extrapolated. As each analyte was present in duplicate on the membrane, intensity values were averaged and the background value was deducted (Supplementary Table 1). The intensity values of the 21-mo-old mice were then divided by the intensity values of 3-mo-old mice to determine the age-related fold-change in analytes for a specific sex group. Additionally, the intensity values of the female mice were then divided by the intensity values of the male mice to determine the sex-specific fold-change in analytes for a specific age group. Each data plot represents pooled samples from 3 mice per age group for each sex.

### 2.5 Enzyme-linked immunosorbent assay

The concentration of CCL21 (DY457) and CXCL13 (DY470) in the PLF and TNFα (DY410) and IL-6 (DY406) in the serum was quantified using enzyme-linked immunosorbent assay (ELISA) (all from Bio-Techne) according to the manufacturer's instructions. For the PLF, neat samples were added in duplicate to the 96-well plate. For the serum samples, singular neat samples were added to the 96-well plate due to limited serum. OD_450_ values were determined using the BioTek Synergy HT plate reader. Background signal was deducted from the OD_450_ values of the samples, standard curves were generated for each chemokine/cytokine, and the concentration of each chemokine/cytokine was then interpolated for the PLF and serum samples. Data are presented as pg/mL.

### 2.6 Statistical analysis

Data were analyzed using GraphPad v8.4.3 Prism (GraphPad Software) and presented as mean ± SEM for n mice per group for n independent experiments. Normality was assessed using Shapiro-Wilk test. Univariate analysis was performed using unpaired *t* test. Multivariate analysis was performed using analysis of variance, with Bonferroni posttest. *P* < 0.05 was deemed statistically significant.

## 3 Results

### 3.1 Leukocyte trafficking to the naïve peritoneal cavity is dysregulated by aging in a sex-specific manner

**Fig. 1.**
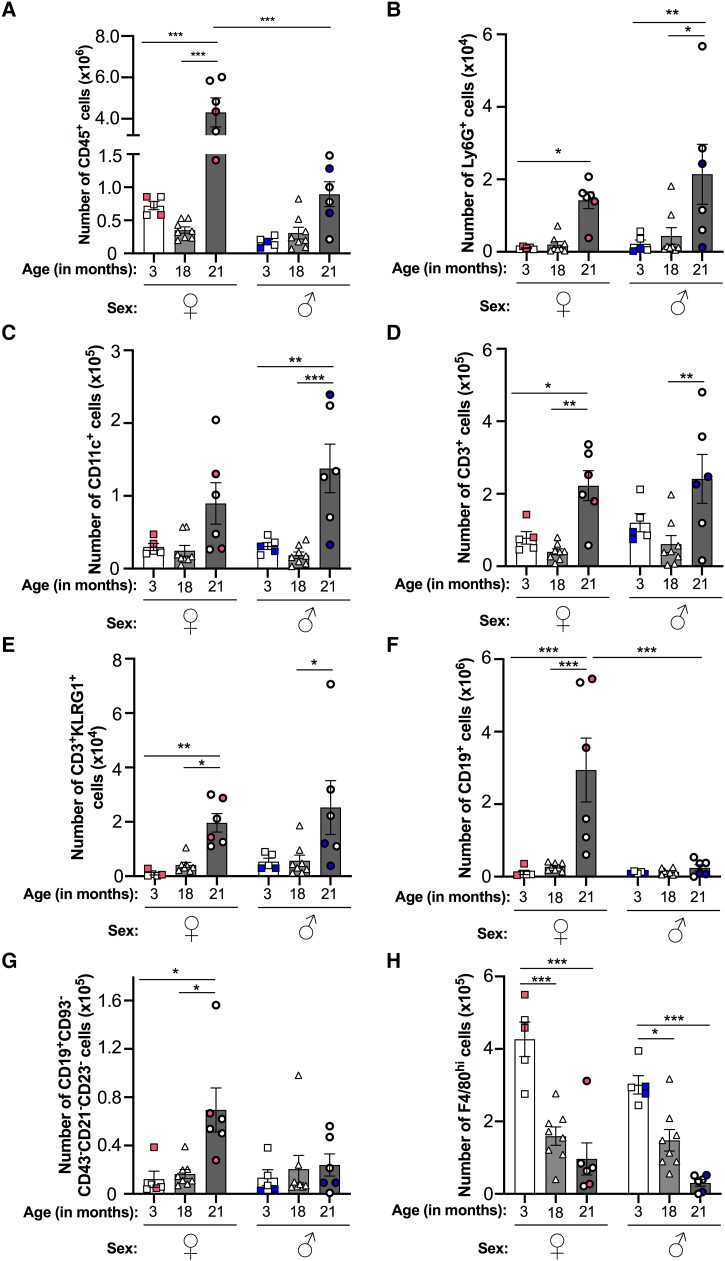
Baseline trafficking of leukocytes into the resting peritoneum is dysregulated by aging in a sex-specific manner. Total number of peritoneal (A) CD45^+^ leukocytes, (B) Ly6G^+^ neutrophils, (C) CD11c^+^ DCs, (D) CD3^+^ T cells, (E) CD3 ^+^ KLRG1^+^ senescent T cells, (F) CD19^+^ B cells, (G) CD19 ^+^ CD93^−^CD43^−^CD21/35^−^CD23^−^ ABCs, and (H) F4/80^hi^ macrophages from resting 3-, 18-, and 21-mo-old female (♀) and male (♂) C57Bl6 mice were quantified using flow cytometry. Analysis of variance showed a significant effect of age on the number of each leukocyte subset (*P* < 0.01) and of sex on the number of leukocytes (*P* < 0.001), macrophages (*P* < 0.01), and B cells (*P* < 0.05) cells. Data are presented as mean ± SEM using n = 5, n = 8, and n = 6 for 3-, 18-, and 21-mo-old mice, respectively, from n = 2 independent experiments per age group for each sex. Color-coded data points indicate independent experiments per age group. *P < 0.05, **P < 0.01, and ***P < 0.001 by Bonferroni multiple comparison posttest.

In order to determine whether aging or biological sex affects homeostatic leukocyte trafficking processes, we initially investigated the leukocyte composition of the peritoneal cavity in young (3-mo), middle aged (18-mo) and old (21-mo) noninflamed mice for both sexes. Low numbers of CD45^+^ leukocytes were detected in the naïve peritoneal cavity of 3- and 18-mo-old mice, and these were comparable across both sexes (Fig. 1A). Aging (above 21 mo) increased the accumulation of CD45^+^ leukocytes within the cavity in both sexes, although this was only significant within the female group (Fig. 1A). Interestingly, significantly higher numbers of CD45^+^ leukocytes were seen in the peritoneum of aged female mice compared with aged male mice (Fig. 1A), indicating age-associated sexual dimorphism in leukocyte trafficking to the cavity under homeostatic conditions.

Further analysis of leukocyte subsets revealed similar age-related trends for peritoneal Ly6G^+^ neutrophils (Fig. 1B), CD11c^+^ dendritic cells (DCs) (Fig. 1C), CD3^+^ T cells (Fig. 1D), and CD3 ^+^ KLRG1^+^ senescent T cells (Fig. 1E)—in which numbers for these subsets were all significantly elevated in the 21-mo-old mice and were broadly comparable between the sexes when compared with 3-mo-old mice. Similar trends were also seen for CD3^+^ T cell subsets, including CD3 ^+^ CD8^+^ T cells, CD8 ^+^ CD62L ^+^ CD44^+^ central memory T cells, and CD8 ^+^ CD62L^−^CD44^+^ effector memory T cells (Supplementary Fig. 3). Interestingly, we observed significantly more Ly6G^+^ neutrophils and CD11c^+^ DCs in male mice at 21 mo-old compared with their 18-mo-old counterparts (Fig. 1B and C). In contrast, CD3^+^ T cell (Fig. 1D) numbers, including CD3 ^+^ KLRG1^+^ senescent T cell (Fig. 1E) and CD8 ^+^ CD62L^−^CD44^+^ effector memory T cell (Supplementary Fig. 3) numbers, were higher in 21-mo-old female mice compared with 18-mo-olds. Other peritoneal leukocyte subsets displayed different age-related trends. Significantly more CD19^+^ B cells and CD19 ^+^ CD93^−^CD43^−^CD21/35^−^CD23^−^ age-associated B cells (ABCs) were detected in the peritoneum of 21-mo-old female mice compared with 3- and 18-mo-old mice (Fig. 1F and G). Interestingly, we observed no age-associated change in these populations in male mice, indicating sexual dimorphism in the trafficking of B cells and ABCs in aged female mice. In contrast to the previous populations, peritoneal F4/80^hi^ macrophages significantly declined with age for both sexes, with significant reductions seen at both 18 and 21 mo (Fig. 1H). Altogether, these observations indicate that aging skews the peritoneal environment toward a proinflammatory state that supports the recruitment of neutrophils, antigen-presenting cells (DCs, B cells), and T cells, including senescent T cells, into the cavity, which may exacerbate the inflammatory environment even further. Moreover, the aging peritoneal environment appears to promote tissue resident macrophage egress from the cavity, which is indicative of the proinflammatory state of the tissue.

### 3.2 Aging modulates the composition of leukocytes in the blood and lymphoid organs in the steady state, indicating leukocyte mobilization

**Fig. 2.**
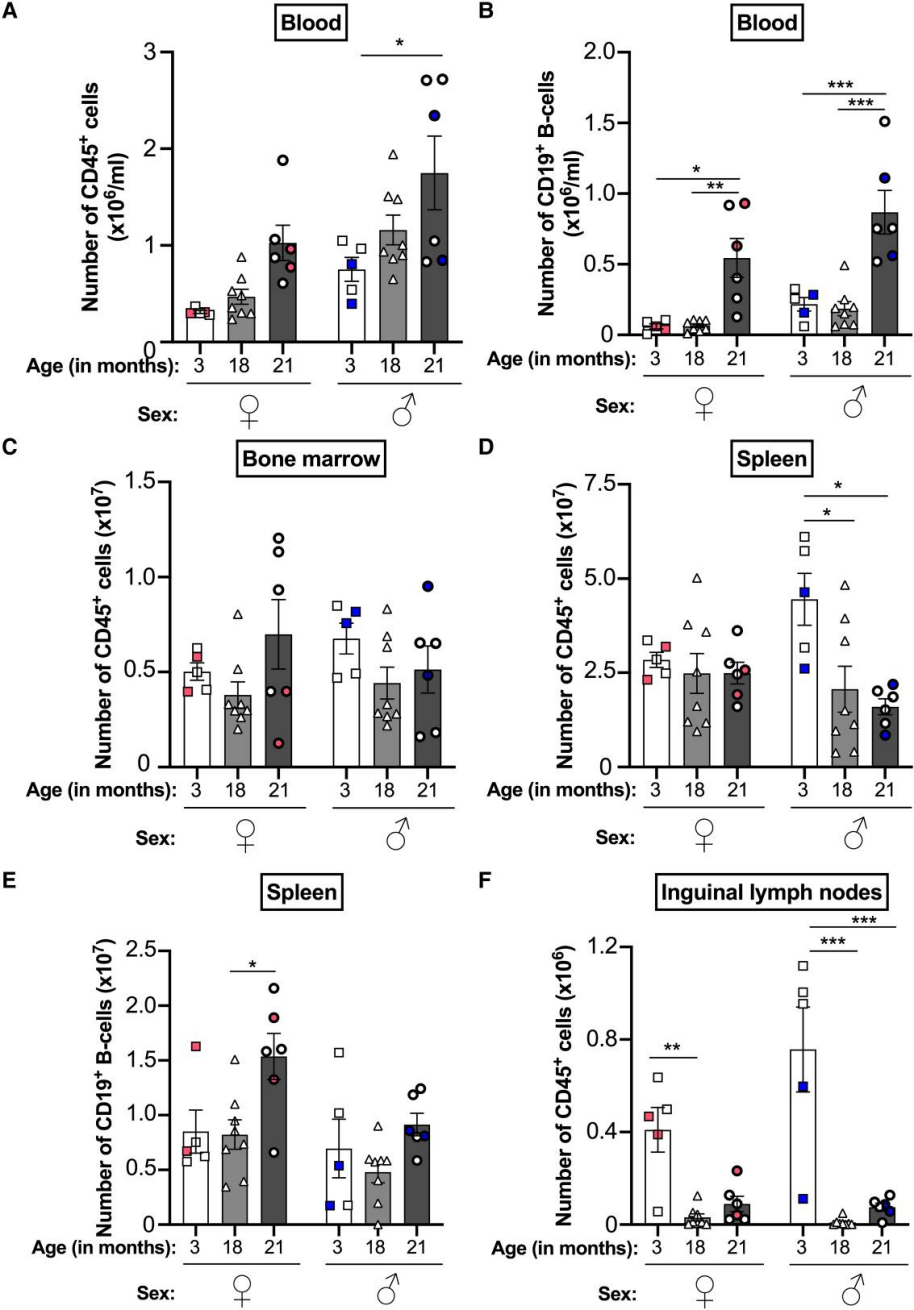
Leukocyte trafficking through blood and lymphoid organs are altered with age, displaying sexual dimorphism. (A, C, D, F) CD45^+^ leukocytes and (B, E) CD19 B cells within the (A, B) blood, (C) bone marrow, (D, E) spleen, and (F) iLNs of naïve 3-, 18- and 21-mo-old female (♀) and male (♂) C57Bl6 mice were quantified using flow cytometry. Analysis of variance showed a significant effect of age on the number of leukocytes in the blood, spleen, and iLNs (*P* < 0.05), and of sex on leukocyte numbers in the blood (*P* < 0.001). Analysis of variance analysis also showed a significant effect of age and sex on the number of B cells in the blood and spleen (*P* < 0.01). Data are presented as mean ± SEM using n = 5, n = 8, and n = 6 for 3-, 18-, and 21-mo-old mice, respectively, from n = 2 independent experiments per age group for each sex. Color-coded data points indicate independent experiments per age group. *P < 0.05, **P < 0.01, and ***P < 0.001 by Bonferroni multiple comparison posttest.

The age-associated changes in leukocyte trafficking into the peritoneal cavity may simply mirror the absolute numbers of these subpopulations circulating in the blood as a consequence of inflammaging. Indeed, circulating levels of TNFα and IL-6 increased with age; however, this was only statistically significant for 21-mo-old female mice (Supplementary Fig. 4) and not for male mice. While age tended to increase circulating numbers of CD45^+^ leukocytes, this was only statistically significant in male mice (Fig. 2A). The numbers of CD19^+^ B cells and Ly6G^+^ neutrophils were also significantly increased in the blood of 21-mo-old male and female mice compared with 3- and 18-mo-old animals (Fig. 2B and Supplementary Fig. 5A) and broadly corresponded with increased numbers of these leukocytes within the aged peritoneal cavity. In contrast, increased circulating numbers of CD11c^+^ DCs were observed in 21-mo-old male mice but not in female mice when compared with 3- and 18-mo-old mice (Supplementary Fig. 5B), which corresponded with increased CD11c^+^ DC numbers in the peritoneal cavity of these mice. It is therefore possible that the age-related increase in these leukocyte populations within the peritoneal cavity may be due to the extravasation of these leukocytes from the blood.

Aging is known to reduce the pool of immune cells within lymphoid tissues, with reduced numbers of B cells in the bone marrow,^25^ T cells in the thymus ^26^ and both cell types in the spleen and LNs^27^ all reported. For example, the T cell population within the iLNs was reported to be reduced by approximately 40% in older (18 to 21 mo) mice compared with young (2 to 3 mo) mice.^28^ As such, we investigated the effect of age and sex on the composition of leukocytes within the lymphoid tissues of the bone marrow, spleen, and iLNs. We observed no difference in CD45^+^ numbers in the bone marrow of mice with age or with sex (Fig. 2C). We observed a significant reduction in CD19^+^ B cell numbers and increase in CD11c^+^ DC numbers within the bone marrow of 21-mo-old female mice, but not in male mice, compared with 3-mo-old animals (Supplementary Fig. 6A and B). While the numbers of CD45^+^ cells remained unchanged with age in the spleens of female mice, we observed a significant age-associated reduction in the number of these cells in the spleens of male mice at 18 and 21 mo-old compared with 3-mo-old animals (Fig. 2D). Aging resulted in reduced CD3^+^ T cell numbers within the spleens of male and female mice (Supplementary Fig. 7A). In contrast, splenic CD19^+^ B cell, CD11c^+^ DC, and F4/80^hi^ macrophage numbers were all increased in 21-mo-old female mice compared with their young counterparts (Fig. 2E and Supplementary Fig. 7B and C). Surprisingly, we observed an almost complete loss of CD45^+^ cells, including CD3^+^ T cells, CD19^+^ B cells, and CD11c^+^ DCs, within the iLNs at 18- and 21 mo-old mice compared with 3-mo-old mice for both sexes (Fig. 2F and Supplementary Fig. 8A to C). Collectively, these data indicate that aging dysregulates leukocyte trafficking through secondary lymphoid tissues, except the bone marrow, and is sexually dimorphic for some tissues (e.g., spleen).

### 3.3 Local chemokine levels are increased in the aging peritoneum

**Fig. 3.**
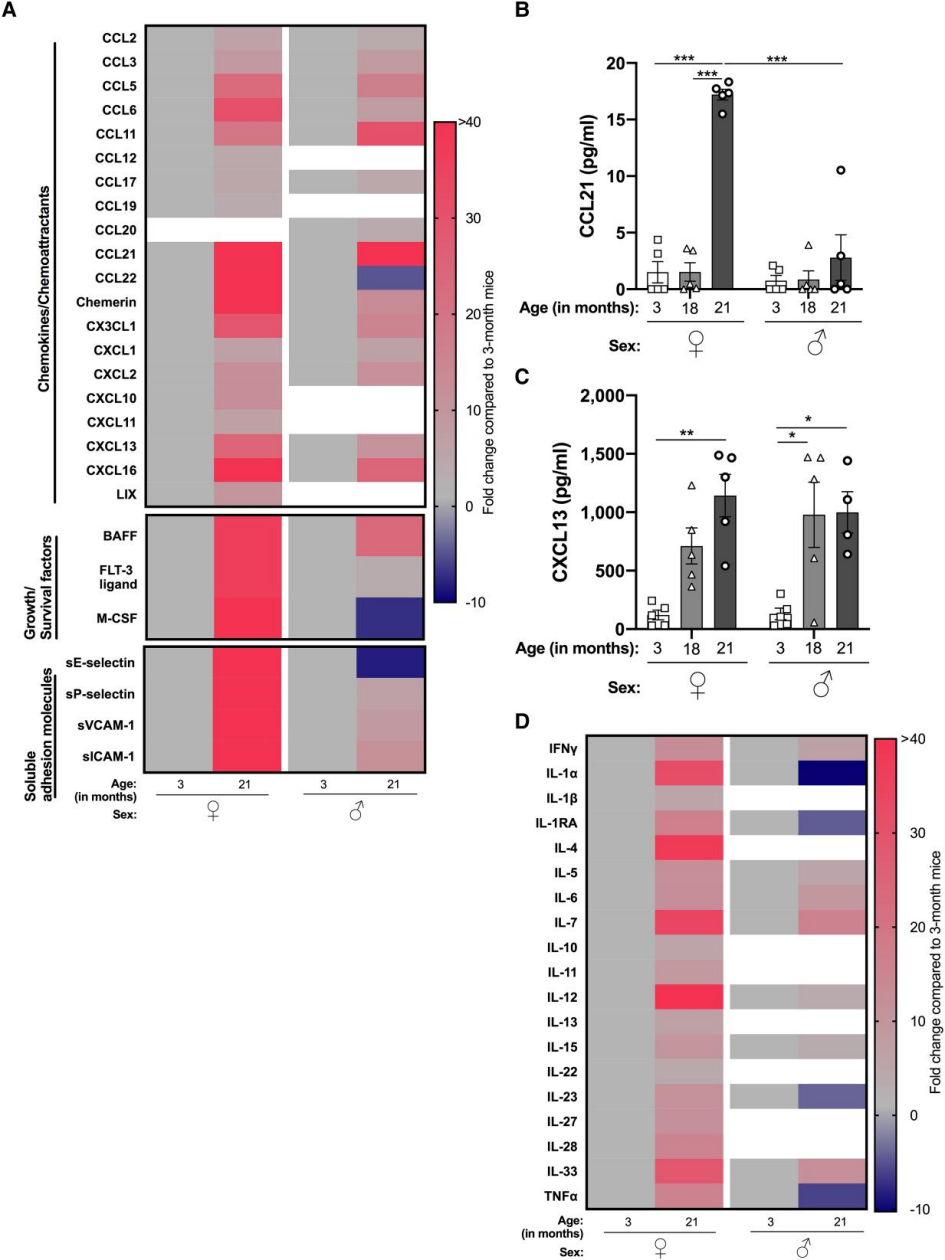
Aging skews the peritoneal secretome toward a proinflammatory microenvironment. An abundance of proinflammatory mediators in the peritoneal fluid of naïve 3- and 21-mo-old female (♀) and male (♂) C57Bl6 mice were analyzed using a cytokine array. Fluids from 3 mice per age group for each sex were pooled, in which n = 1. Heatmaps depict the fold change in (A) chemokines/chemoattractants, growth/survival factors, and soluble adhesion molecules, with (D) cytokines of aged mice relative to young mice for each sex. Analytes with <3-fold change are represented by white squares. Quantification of (B) CCL21 and (C) CXCL13 in the peritoneal lavage fluid of 3-, 18-, and 21-mo-old female and male wild-type mice by ELISA. Analysis of variance showed a significant effect of age (*P* < 0.001) and sex (*P* < 0.001) on the concentration of CCL21 in the peritoneal fluid and of age (*P* < 0.001), but not sex, on the concentration of CXCL13. Data are presented as mean ± SEM using n = 4 to 5 per age group, from n = 1 independent experiment. *P < 0.05, **P < 0.01, and ***P < 0.001 by Bonferroni multiple comparison posttest.

Next, we examined whether the age-related and sex-specific differences in leukocyte recruitment to the peritoneal cavity under homeostatic conditions could be explained by variations in the levels of chemokines/chemoattractants, leukocyte survival/growth factors, and soluble adhesion molecules within the PLF, reminiscent of previous studies.^15,29^ Indeed, we observed a ≥3-fold increase in the levels of chemokines in the peritoneal cavity of aged male and female mice compared with young mice, with these age-associated changes being more pronounced in female mice (Fig. 3A and Supplementary Fig. 9). Interestingly, some chemokines (CCL12, CCL19) were restricted to age-associated increases in their expression in a sex-specific manner. For example, a ≥3-fold increase in the expression of CCL12, CCL19, CXCL10, CXCL11, and LIX was only observed in the PLF of aged female but not aged male mice compared with young animals (Fig. 3A). Conversely, there was a ≥3-fold increase in the levels of CCL20 in aged male mice compared with young males that was not evident in aged females. Interestingly, CCL22 levels were increased >40-fold in aged female mice compared with young females but was reduced >4-fold in aged male mice compared with their young counterparts. Despite the sex-specific differences, these data collectively suggest that aging is associated with a dramatic increase in chemokine/chemoattractant levels within the peritoneal cavity, which may support increased leukocyte recruitment. To validate the array data, we quantified the concentrations of the B cell chemoattractants, CCL21 and CXCL13. CCL21 was significantly elevated in the PLF of female mice at 21 mo compared with female mice at 3 and 18 mo, and also compared with 21 mo-old male mice (Fig. 3B). CXCL13 was significantly increased in the PLF of 21-mo-old female and male mice compared with 3- and 18-mo-old mice (Fig. 3C). Collectively, these data may explain the elevated levels of B cell recruitment to the cavity of aged female mice.

Other soluble mediators, such as growth factors and survival signals, could also be responsible for the alterations in peritoneal leukocyte numbers that we have observed in 21-mo-old mice. We saw a ≥ 3-fold increase in the levels of BAFF (a B cell survival factor) and FLT-3 (a hematopoietic cell growth/survival factor) in the PLF of aged male and female mice compared with young animals, although the age-associated change in expression was more pronounced for the female mice (Fig. 3A). Interestingly, we observed a >40-fold increase in the levels of macrophage colony-stimulating factor (M-CSF) (a monocyte/macrophage differentiation/survival factor) in aged female mice compared with young female mice, while it was reduced >7-fold in aged male mice compared with young male mice (Fig. 3A). There was a ≥3-fold increase in the abundance of soluble adhesion molecules P-selectin, ICAM-1, and VCAM-1 in the PLF of 21-mo-old male and female mice compared with young mice, although the increase was again more striking for the female mice (Fig. 3A). Levels of soluble E-selectin were increased >40-fold in aged female mice, while >8-fold lower levels were seen in aged male mice when compared with the young sex-matched counterparts (Fig. 3A). Additionally, we observed a ≥ 3-fold increase in peritoneal expression of soluble proteolytic enzymes (e.g., MMP-2 and MMP-3 for aged female mice) but a ≥ 10-fold decrease in the level of these enzymes in the PLF of aged male mice compared with young mice (Supplementary Fig. 10). Collectively, these data demonstrate that aging is associated with increased levels of immune cell growth and survival factors, soluble adhesion molecules, and proteolytic enzymes within the peritoneal cavity.

### 3.4 Aging skews the peritoneal secretome toward a proinflammatory environment

To understand whether the age-associated increase in peritoneal leukocyte numbers corroborated a proinflammatory environment, we assessed the relative abundance of pro- and anti-inflammatory mediators in the PLF of 21-mo-old male and female mice compared with 3-mo-old mice using a cytokine array (Fig. 3D). We observed a ≥ 3-fold increase in the proinflammatory cytokines, including IFNγ, IL-5, IL-12, IL-15, and IL-33, in the peritoneal cavity of aged male and female mice compared with young mice. Aged female PLF also had a ≥3-fold increase in the levels of proinflammatory mediators IL-1α, IL-1β, IL-23, TNFα, IL-22, and IL-27 when compared with young female mice (Fig. 3D). Interestingly, pleiotropic cytokines IL-6 and IL-7 were increased in the PLF of aged mice compared with young mice, independent of sex (Fig. 3D). Collectively, these data suggest that aging alters the inflammatory environment of the peritoneal cavity in a sex-specific manner and, overall, skews it toward a more proinflammatory and prosurvival (e.g., IL-7, IL-15) phenotype (i.e., more pronounced in females).

### 3.5 Aging is associated with increased angiogenesis and reduced integrity of the peritoneal membrane vasculature

**Fig. 4.**
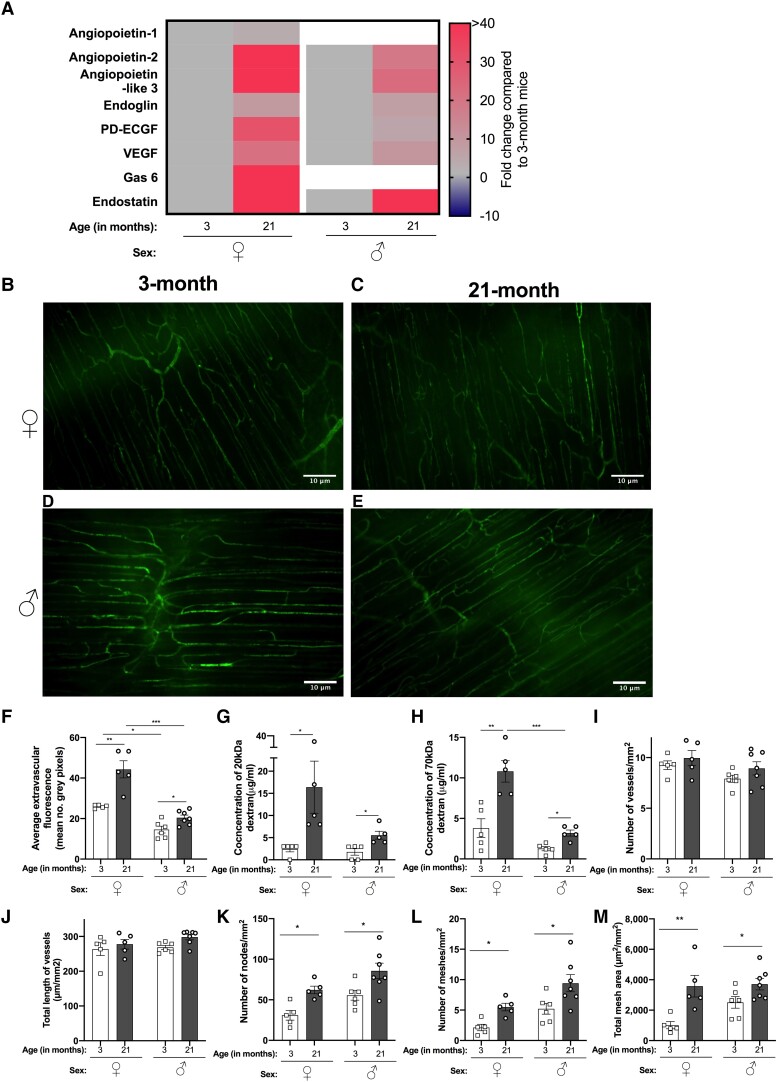
Aging was associated with increased permeability and modulated structure of the peritoneal membrane vasculature. (A) Relative abundance of angiogenic factors in the peritoneal fluid of naïve 3- and 21-mo-old female (♀) and male (♂) C57Bl6 mice were analyzed using a cytokine array. Fluids from 3 mice per age group were pooled, in which n = 1. Heatmaps depict the fold change in cytokines and angiogenic factors of aged mice relative to young mice for each sex group. The vasculature of the peritoneal membrane from (B, D) young (3 mo) and (C, E) aged (21 mo) (B, C) female and (D, E) male mice were imaged using intravital microscopy. Images were quantified to assess (F) the leakage of fluorescent dextran into the extravascular space, expressed as mean number of gray pixels; (I) average number of vessels; (J) total length of vessels; (K) number of nodes; (L) number of meshes; and (M) total mesh area all expressed per mm^2^. Quantification of the concentration of (G) 20 kDa and (H) 70 kDa fluorescent dextran within the peritoneal lavage fluid postimaging. Data are mean ± SEM for n = 5 independent experiments, using 5 mice per age group. *P < 0.05, **P < 0.01, ***P < 0.001 by Bonferroni multiple comparison test. Scale bar = 10 μm.

To determine if changes to the peritoneal membrane vasculature contributed to increased leukocyte recruitment to the aged peritoneal cavity, we assessed the relative abundance of pro- and antiangiogenic factors in the PLF of 21-mo-old male and female mice compared with 3-mo-old animals using a cytokine array. We observed increased levels of several proangiogenic factors (angiopoietin-2, angiopoietin-like 3, endoglin, PD-ECGF, VEGF) and 1 antiangiogenic factor (endostatin) in the PLF of 21-mo-old mice compared with 3-mo-old mice, with the most prominent changes occurring in the aged female mice (Fig. 4A). We also observed an increase in the proangiogenic factors angiopoietin-1 and Gas6 in the PLF of 21-mo-old female mice compared with 3-mo-old mice, but these levels were unchanged in the PLF of male mice (Fig. 4A). Collectively, these data suggest that age-related changes occur to the angiogenic processes of the peritoneal membrane vasculature, which are more pronounced in the females.

In agreement with published literature,^30,31^ our data indicate age-related changes to vasculature integrity were linked to angiogenic processes; therefore, we examined this further in the context of leukocyte trafficking into the peritoneum. We analyzed the vasculature structure in the peritoneal membrane and its integrity/permeability following perfusion of fluorescently labelled dextrans (20 and 70 kDa) in 3- and 21-mo-old female and male mice (Fig. 4B to E). In young mice, the peritoneal membrane vasculature appeared organized (Fig. 4B and D) with minimal extravascular leakage of fluorescent dextran (Fig. 4F). By contrast, the vasculature in old mice appeared disorganized with more vessel circuits (looping structures) (Fig. 4C and E) and loss of integrity, as evidenced by a significant increase in fluorescence dextran within the extravascular space compared with the young mice, which was more pronounced in the females (Fig. 4F). Indeed, quantifying the concentrations of the 20 and 70 kDa fluorescent dextran in the PLF revealed significantly more of each dextran in the peritoneum of aged mice, particularly aged female mice, compared young mice (Fig. 4G and H), indicating increased vascular permeability in the aged animals. Together, these data suggest that aging reduces the integrity of the peritoneal membrane vasculature, which may facilitate increased leukocyte trafficking into the peritoneal cavity.

Further analysis of the morphological structure of the vascular network in the peritoneal membrane revealed no significant difference in the number of vessels or total length of the vessels per mm^2^ between young and aged mice (Fig. 4I and J). Interestingly, we observed a significant increase in the number of points of vessel bifurcation (termed nodes), vessel circuits (termed meshes), and total mesh area per mm^2^ in aged mice compared with young mice (Fig. 4K to M). Increased numbers of vessel circuits within the peritoneal membrane vasculature in combination with increased vessel permeability may offer leukocytes more time to extravasate into the cavity in the steady state, contributing to age-related changes in peritoneal leukocyte composition.

## 4 Discussion

Homeostatic leukocyte trafficking is key to maintain tissue homeostasis and immunosurveillance.^13^ Although growing evidence supports the notion that the aging process affects leukocyte trafficking patterns during inflammation (reviewed by Hopkin et al.),^9^ a limited number of studies have investigated the impact of chronological aging on homeostatic leukocyte trafficking, and even fewer have considered sexual dimorphism within these age-related changes.^32–35^ Here, we found that aging was associated with significantly increased numbers of leukocytes and proinflammatory mediators in the peritoneal cavity of mice in the steady state. Importantly, these age-related changes occurred in a sex-specific manner, with increased vascular changes observed in female mice.

Peritonitis is a well-established and widely used model to study leukocyte trafficking during acute inflammation, in which the effects of age^36,37^ and sex^15,38,39^ on leukocyte recruitment to the peritoneal cavity in this model have been investigated. But studies investigating age-related changes to peritoneal leukocyte populations in the steady state are often limited to male mice.^7,40^ In aged mice, we observed increased numbers of peritoneal leukocytes, including neutrophils and B cells, and reduced macrophage levels, which is in line with Mogilenko et al.,^7^ who reported a similar profile in the peritoneal cavity of aged (17 to 24 mo) male C57Bl6 mice compared with young (3 to 6 mo) using single-cell RNA sequencing. Moreover, Linehan et al.^40^ also described reduced frequencies of peritoneal macrophages in aged (15 to 20 mo) male C57Bl6 mice compared with young mice (<3 mo). We extend and report for the first time the impact of sexual dimorphism in the context of aging at the steady state. Older female mice displayed an exacerbated increase in peritoneal leukocyte numbers, especially B cells, compared with aged male mice, suggesting that females may be more susceptible to age-related changes in leukocyte trafficking patterns into the peritoneal cavity.

Increased numbers of peritoneal leukocytes in aged mice fits the paradigm of inflammaging, whereby the development of chronic inflammation during the aging process alters the inflammation status of peripheral tissues.^41–43^ We found a significant age-associated reduction in the number of leukocytes residing in the spleen and iLNs and an increase in circulating leukocytes, suggesting mobilization of leukocytes from lymphoid tissues. The structure and function of LNs are influenced by the aging process, including the leukocyte composition of the tissue (reviewed by Turner and Mabbott).^27^ Here, we report an age-related reduction in the number of B cells within the iLNs of 21-mo-old mice compared with 3-mo-old mice. In contrast, Turner and Mabbott^28^ reported an increase in B cells in LNs from aged female C57Bl6 mice compared with young mice, but these analyses were obtained from cervical, axillary, brachial, and inguinal LNs pooled into a single-cell suspension, which confounds interpretation. Although no direct comparisons have been made for male mice, studies utilizing rats suggest that aging has a similar impact on architecture of LN in male animals, including distorted tissue structure and cellular composition and reduced numbers of germinal center reactions.^44,45^

Leukocyte mobilization from lymphoid tissues into the circulation was described by Kay et al.,^15^ whereby increased numbers of circulating Gr1^high^ neutrophils, coupled with reduced levels in the spleen, correlated with increased recruitment of neutrophils into the peritoneal cavity upon induction of peritonitis. While acute inflammation (e.g., peritonitis) is not comparable to age-associated chronic inflammation,^46^ our data from steady-state aged animals mirrors inflammation induced leukocyte trafficking between lymphoid tissue and the peritoneal cavity, thus suggesting that aging influences the dynamics of trafficking between these compartments. Indeed, increased numbers of B cells in the peritoneal cavity of aged female mice could be the result of age-related changes to B cell trafficking patterns, indicated by reduced B cell numbers in lymphoid tissues and increased B cell numbers in the circulation. That said, other factors could also potentially explain these findings (e.g., aging may influence leukocyte proliferation, apoptosis, or retention rates within the peritoneal cavity). Although there is currently no specific literature on age-related changes to proliferation/apoptosis rates of peritoneal leukocytes, there is an abundance of evidence supporting age-related changes to leukocyte proliferation^47–49^ and apoptosis^49–52^ rates across multiple tissues in humans and mice. For example, naïve CD4^+^ T cells isolated from the spleens of older (18 to 20 mo) C57Bl6 mice exhibited increased rates of apoptosis in response to antigen stimulation ex vivo compared with those isolated from young (2 to 3 mo) mice.^53^ Whether aging specifically alters peritoneal leukocyte proliferation and apoptosis rates remains to be answered.

Published datasets regarding the sex-specific differences in peritoneal leukocyte populations of noninflamed mice have primarily utilized young mice and are usually a byproduct of models of inflammation.^15,38,39^ In agreement with previous studies,^15,38^ we report increased leukocyte numbers, including increased F4/80^hi^ macrophages, in the peritoneal cavity of 3-mo-old female mice compared with 3-mo-old male mice. Interestingly, Scotland et al.^38^ reported higher numbers of peritoneal leukocytes in young (<5 mo) female C57Bl6 mice compared with young male mice, and importantly, these changes were not due to sex differences in leukocyte apoptosis or proliferation rates, but rather were attributed to the effects of sex steroids. Indeed, ovariectomized female mice had comparable numbers of peritoneal leukocytes to young male mice, suggesting that female sex hormones play a direct role in the regulation of leukocyte trafficking to the peritoneal cavity. Both estrogen and progesterone reportedly suppress endothelial activation (e.g., estrogen downregulates VCAM-1 expression on TNFα-activated endothelial cells),^54–58^ while progesterone inhibits IL-6, IL-8, CXCL2, and CXCL1 production by lipopolysaccharide-stimulated human umbilical vein endothelial cells in vitro.^55^ In the steady state, levels of soluble ICAM-1 and VCAM-1 within serum were reportedly higher in the early phases of the menstrual cycle, in which circulating levels of estrogen and progesterone are at their lowest, and correlated with reduced numbers of circulating monocytes.^59^ Therefore, sex hormones likely play a role in regulating leukocyte trafficking in young mice but not in aged mice due to the age-related decline in circulating sex hormone levels (reviewed by Horstman et al).^60^

Increased levels of chemokines and soluble adhesion molecules within the aging peritoneal cavity may have facilitated the increased trafficking of various leukocytes that we observed. The approach used was an unbiased analysis of pooled samples, which may be skewed by individual mice and therefore requires validating using alternative methodologies. Indeed, ELISA analysis confirmed increased levels of the B cell chemoattractants CCL21 and CXCL13 in the PLF of aged female mice compared with young, offering a potential mechanism of action for increased B cell recruitment to the aging cavity. The changes in leukocyte numbers observed here could also be caused by an age-related increase in the survival and growth of certain leukocyte populations, such as B cells, mediated by BAFF and the FLT-3 ligand, which were increased within the aging cavity. Indeed, Amezcua Vesely et al.^61^ demonstrated that BAFF treatment of peritoneal B1 cells ex vivo protected the B cells from FcγRIIb-mediated apoptosis. Whether age-related changes in the composition and number of leukocytes within the peritoneal cavity are a result of increased leukocyte trafficking or altered proliferative/apoptotic rates remains to be clarified.

TNFα and IL-6, 2 of the most extensively studied cytokines released as part of the SASP, are increased in the serum of older adults^62,63^ and older mice^64,65^ and can modulate the expression of adhesion molecules on endothelial cells.^66–69^ Coupled with the increase in circulating TNFα and IL-6 in 21-mo-old female mice, we also observed increases in proteolytic enzymes (e.g., MMP-2 and MMP-3) that may explain increased levels of soluble adhesion molecules. Several studies to date have reported increased abundance of soluble ICAM-1 and VCAM-1 in the circulation of older (>50 yr) adults compared with younger (<50 yr) adults.^70–74^ The sex-specific differences in circulating soluble adhesion molecule expression have not been extensively studied, but some reports indicate that young (<53 yr) women have lower levels of soluble ICAM-1, VCAM-1 and P-selectin compared with young men in the steady state.^75,76^ Indeed, peritoneal levels of soluble ICAM-1 have been reported to correlate with increased polymorphonuclear cell recruitment to the peritoneal cavity during episodes of bacterial peritonitis in humans, likely due to the shedding of this molecule from activated endothelial cells during the inflammatory response.^29^ Further work is required to determine the sex-specific molecular mechanism governing the cleavage/release of soluble adhesion molecules in aged mice.

Aging resulted in reduced macrophage numbers within the peritoneal cavity of aged male and female mice compared with young mice. However, levels of M-CSF, a cytokine that promotes macrophage proliferation and differentiation,^77^ were only reduced in the peritoneal cavity of aged male mice. In fact, we observed a >40-fold increase in M-CSF levels in the peritoneal cavity of aged female mice. This could be explained by increased levels of proinflammatory mediators, such as TNFα (>15-fold), in the peritoneal cavity of aged female mice compared with aged male mice that may counteract the proliferation-inducing effects of M-CSF and induce macrophage apoptosis. It is important to bear in mind that the levels of M-CSF were obtained from pooled analysis and would need further validation for firm conclusions to be drawn. Indeed, TNFα can induce apoptosis of monocyte-derived macrophages in vitro when nuclear factor κB signaling is suppressed.^78^ Collectively, these data suggest that the maintenance of resident macrophage populations within the aging peritoneal cavity is sexually dimorphic.

Although we did not observe any changes in the vascular density of the peritoneal membrane with aging, we did observe increased levels of several proangiogenic factors within the aged cavity, and this was associated with altered vasculature structure. In line with previous studies looking at the cerebral vasculature in humans with age,^79^ we saw an increased number of vessel bifurcations/branch points and vessel circuits in the aged vasculature compared with young vasculature, which may be due to the increased levels of proangiogenic factors within the cavity. Aging adversely affects angiogenesis and vessel structure,^80^ primarily thought to be due to a reduction in the production of VEGF by macrophages and endothelial cells.^81,82^ Contrasting these observations, we found elevated levels of VEGF within the aged cavity, which may facilitate local angiogenesis in the cavity. It is quite possible that the impact of aging on angiogenic processes, and therefore the vasculature structure, occurs in a tissue-specific manner. Despite the increases in complex vessel structures in our aged mice, these displayed reduced integrity leading to increased leakage of fluorescent dyes into the peritoneal cavity, particularly for the aged female mice. Our data agree with several studies that have demonstrated reduced vascular integrity with age.^30,31,83–85^ It is quite possible that the age-related proinflammatory state of the peritoneal cavity (increased cytokines, chemokines etc.) encourages increased vascular permeability. Indeed, Owen-Woods et al.^86^ reported increased trafficking of neutrophils into the cremaster muscle of young (<3 mo) male C57Bl6 mice in response to inflammation-mediated vascular leakage of CXCL1 into the circulation. It is possible that leakage of B cell chemoattractants CXCL13 and CCL21 into the circulation of aged mice promoted B cell recruitment to the peritoneal cavity. In addition, we observed increased trafficking of ABCs to the peritoneal cavity of aged female mice, but not of aged male mice, in the steady state. ABCs are long lived antigen-experienced B cells that accumulate with age^87,88^ and are believed to contribute to the progression of several autoimmune diseases.^89^ It is therefore possible that ABCs within the peritoneal cavity of aged mice could further exacerbate the heightened inflammatory state of this tissue, driving pathological consequences. Additionally, increased vessel circuits within the peritoneal membrane vasculature in combination with reduced vessel integrity may serve as a potential mechanism to explain increased leukocyte trafficking to this tissue in aging.

## 5 Conclusion

Homeostatic leukocyte trafficking is essential to immune function; however, understanding how aging and biological sex affects these processes has largely been overlooked. In this study, we found a striking increase in the number of leukocytes in the peritoneal cavity of aged mice, particularly in female mice, and this was associated with a proinflammatory microenvironment. We demonstrated increased permeability of the peritoneal membrane vasculature, which may support increased leukocyte recruitment to the peritoneal cavity in aging. To conclude, these types of study are required to further our knowledge of the impact that age and sex differences have on immune cell trafficking and function, which will potentially expand our understanding of how these changes drive disease or injury.

## Supplementary Material

qiad053_Supplementary_Data
