# Supplementary material for: Aging modulates homeostatic leukocyte trafficking to the peritoneal cavity in a sex-specific manner
Source: J Leukoc Biol. 2023 Jun 13;114(4):301–14. doi: 10.1093/jleuko/qiad053 (PMC10533226; doi:10.1093/jleuko/qiad053)
Supplement: qiad053_Supplementary_Data [file qiad053_supplementary_data.docx]

**Supplementary Figure 1 – Flow cytometry gating strategy for peritoneal cells**

The flow cytometry gating strategy used to phenotype and quantify leukocytes within the peritoneal exudate of naïve young (3 month), middle-aged (18 month) and aged (21 month) male and female C57Bl6 mice. Gating strategy to define **(A)** beads and single, live CD45^+^ leukocytes. Gating strategy to identify **(B)** F4/80^hi^ macrophages, CD11c^+^ dendritic cells, **(C)** Ly6G^+^ neutrophils, **(D)** SiglecF^+^ eosinophils, **(E)** CD3^+^ T-cells and CD19^+^ B-cells within the leukocyte population. **(F)** T-cells were distinguished based on CD4 and CD8 expression, and then based on CD62L and CD44 expression to identify CD62L^+^CD44^-^ naïve, CD62L^-^CD44^+^ effector memory and CD62L^+^CD44^+^ central memory subsets. **(G)** Regulatory T-cells were identified as CD3^+^CD4^+^CD25^+^ cells. **(H)** Senescent T-cells were identified as CD3^+^KLRG1^+^ cells. **(I)** Age-associated B-cells were identified as CD19^+^CD93^-^CD43^-^CD21^-^CD23^-^ cells.

**Supplementary Figure 2 – Cytokine array using peritoneal fluid from young and aged mice.**

The abundance of pro-inflammatory mediators in the peritoneal fluid of naïve 3- and 21-month female (♀) and male (♂) C57Bl6 mice were analysed using a cytokine array. Fluids from 3 mice per age group for each sex were pooled, where n=1. The four cytokine arrays are shown.

**Supplementary Figure 3 - T-cell subsets in the peritoneum of young and aged wildtype mice**

Total number of peritoneal **(A)** CD4^+^, **(B)** CD8^+^, **(C)** CD4^+^ naive (N), **(D)** CD4^+^ effector memory (EM), **(E)** CD8^+^ N, **(F)** CD8^+^ EM, **(G)** CD8^+^ CM, and **(H)** regulatory T-cells (Tregs) from resting 3, 18, and 21 month female (♀) and male (♂) C57Bl6 mice were quantified using flow cytometry. Data are shown as mean ± standard error of the mean (SEM) for lymphocyte subsets using n=5, n=8 and n=6 for 3-, 18- or 21-month-old mice, respectively, from n = 1-2 independent experiments per age group for each sex.. Statistical analysis performed using two-way ANOVA to determine the effects of sex and age on the number of peritoneal lymphocyte subsets. Only significant data is depicted. *p<0.05, **p<0.01, and ***p<0.001 by Bonferroni multiple comparison post-test.

**Supplementary Figure 4 - Circulating pro-inflammatory mediators in young and aged mice.**

The concentration of (**A**) TNFa and (**B**) IL-6 in the circulation of naive 3-month, 18-month, 21-month female and male mice using ELISA. ANOVA showed a significant effect of age (p<0.001) on circulating TNFa levels, and of age (p<0.001) and sex (p<001) on circulating IL-6 levels. Data are presented as mean ± SEM using n=4-5, n=8 and n=4-6 for 3-, 18- or 21-month-old mice, respectively, from n = 1 independent experiment. *p<0.05 by Bonferroni multiple comparison post-test.

**Supplementary Figure 5 - Innate and adaptive leukocyte population numbers in the blood of young and aged wildtype mice**Total number of circulating **(A)** Ly6G^+^ neutrophils, **(B)** CD11c^+^ DC , **(C)** SiglecF^+^ eosinophils, **(D)** CD19^+^CD93^-^CD43^-^CD21^-^CD23^-^ age-associated B-cells (ABCs), **(E)** CD3^+^ T-cells, and **(F)** CD3^+^KLRG1^+^ senescent T-cells from resting 3, 18, and 21 month female (♀) and male (♂) C57Bl6 mice were quantified using flow cytometry. Data are shown as mean ± standard error of the mean (SEM) for leukocyte populations. Statistical analysis performed using two-way ANOVA to determine the effects of sex and age on the number of circulating leukocytes. Only significant data is depicted.

**Supplementary Figure 6 - Innate and adaptive leukocyte population numbers in the bone marrow of young and aged wildtype mice**

Total number of **(A)** CD19^+^ B-cells, **(B)** CD11c^+^ DC, **(C)** SiglecF^+^ eosinophils, **(D)** Ly6G^+^ neutrophils, **(E)** CD3^+^ T-cells, and **(F)** CD3^+^KLRG1^+^ senescent T-cells in the bone marrow of resting 3, 18, and 21 month female (♀) and male (♂) C57Bl6 mice were quantified using flow cytometry. Data are shown as mean ± standard error of the mean (SEM) for leukocyte populations. Statistical analysis performed using two-way ANOVA to determine the effects of sex and age on the number of leukocytes.

**Supplementary Figure 7 - Innate and adaptive leukocyte population numbers in the spleen of young and aged wildtype mice**Total number of splenic **(A)** CD3^+^ T-cells, **(B)** CD11c^+^ DC, **(C)** F4/80^hi^ macrophages, **(D)** CD19^+^CD93^-^CD43^-^CD21^-^CD23^-^ age-associated B-cells (ABCs), **(E)** SiglecF^+^ eosinophils, and **(F)** CD3^+^KLRG1^+^ senescent T-cells from resting 3, 18, and 21 month female (♀) and male (♂) C57Bl6 mice were quantified using flow cytometry. Data are shown as mean ± standard error of the mean (SEM) for leukocyte populations. Statistical analysis performed using two-way ANOVA to determine the effects of sex and age on the number of splenic leukocytes. Only significant data is depicted.

**Supplementary Figure 8 - Innate and adaptive leukocyte population numbers in the inguinal lymph nodes of young and aged wildtype mice**

Total number of **(A)** CD3^+^ T-cells, **(B)** CD19^+^ B-cells, **(C)** CD11c^+^ DC, **(D)** F4/80^hi^ macrophages, **(E)** SiglecF^+^ eosinophils, and **(F)** CD3^+^KLRG1^+^ senescent T-cells in the inguinal lymph nodes of resting 3, 18, and 21 month female (♀) and male (♂) C57Bl6 mice were quantified using flow cytometry. Data are shown as mean ± standard error of the mean (SEM) for leukocyte populations. Statistical analysis performed using two-way ANOVA to determine the effects of sex and age on the number of leukocytes.

**Supplementary Figure 9 – Sexual dimorphic expression of analytes in the peritoneal cavity**

Abundance of pro-inflammatory mediators in the peritoneal fluid of naïve 3- and 21-month female (♀) and male (♂) C57Bl6 mice were analysed using a cytokine array. Fluids from 3 mice per age group for each sex were pooled, where n=1. Heatmaps depict the fold change in **(A)** chemokines/chemoattractants, growth/survival factors, soluble adhesion molecules, **(B)** cytokines, and angiogenic factors of female mice relative to male mice for each age group. Analytes with <3 fold-change are represented by white squares

**Supplementary Figure 10 – Age-related changes to the expression of enzymes in the peritoneal cavity**

Abundance of enzymes in the peritoneal fluid of naïve 3- and 21-month female (♀) and male (♂) C57Bl6 mice were analysed using a cytokine array. Fluids from 3 mice per age group for each sex were pooled, where n=1. Heatmaps depict the fold change of enzyme abundance in the PLF of **(A)** aged mice relative to young mice for each sex, and **(B)** female mice relative to male mice for each age group. Analytes with <3 fold-change are represented by white squares

**Supplementary Figure 1**


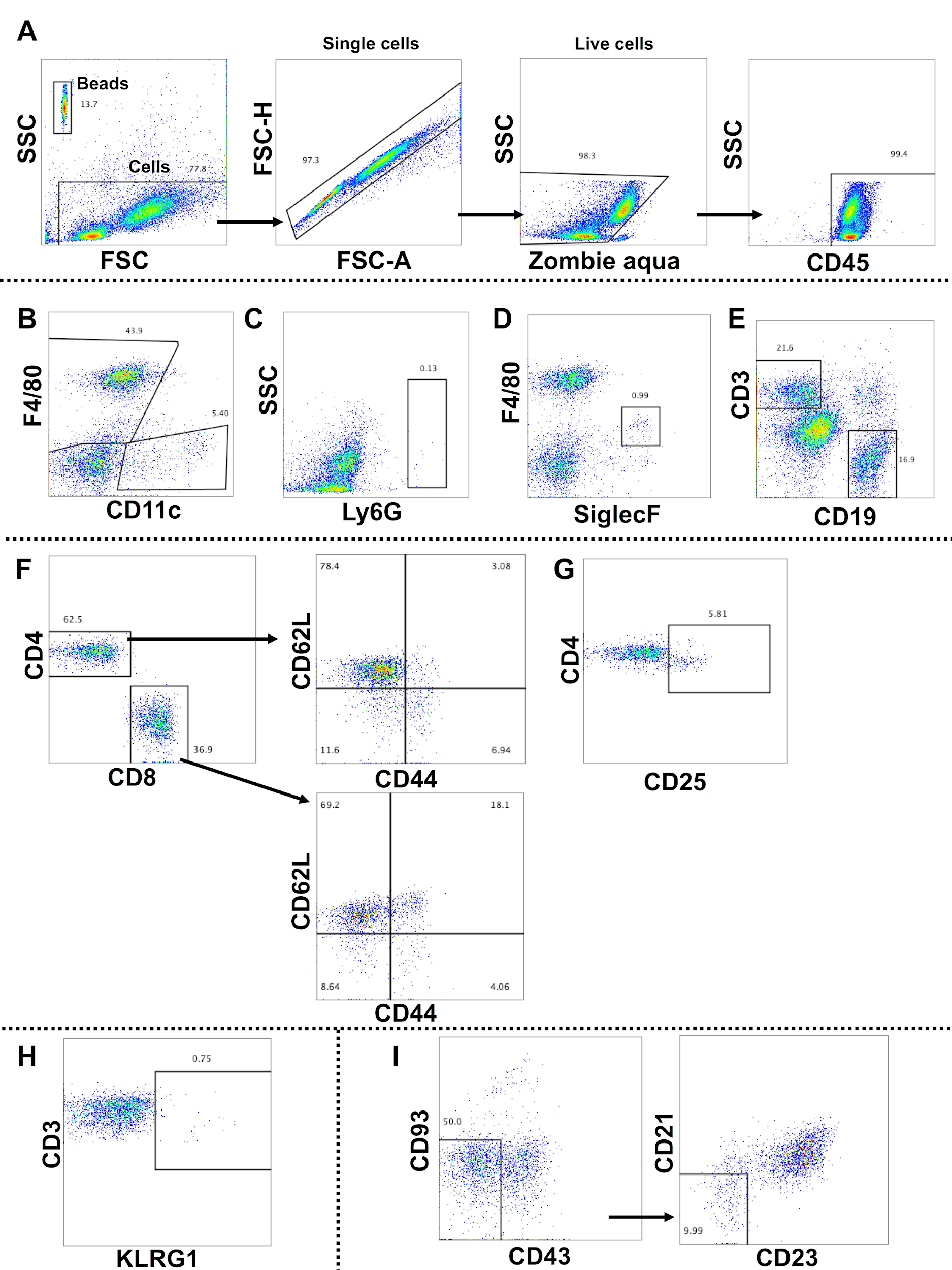


**Supplementary Figure 2**


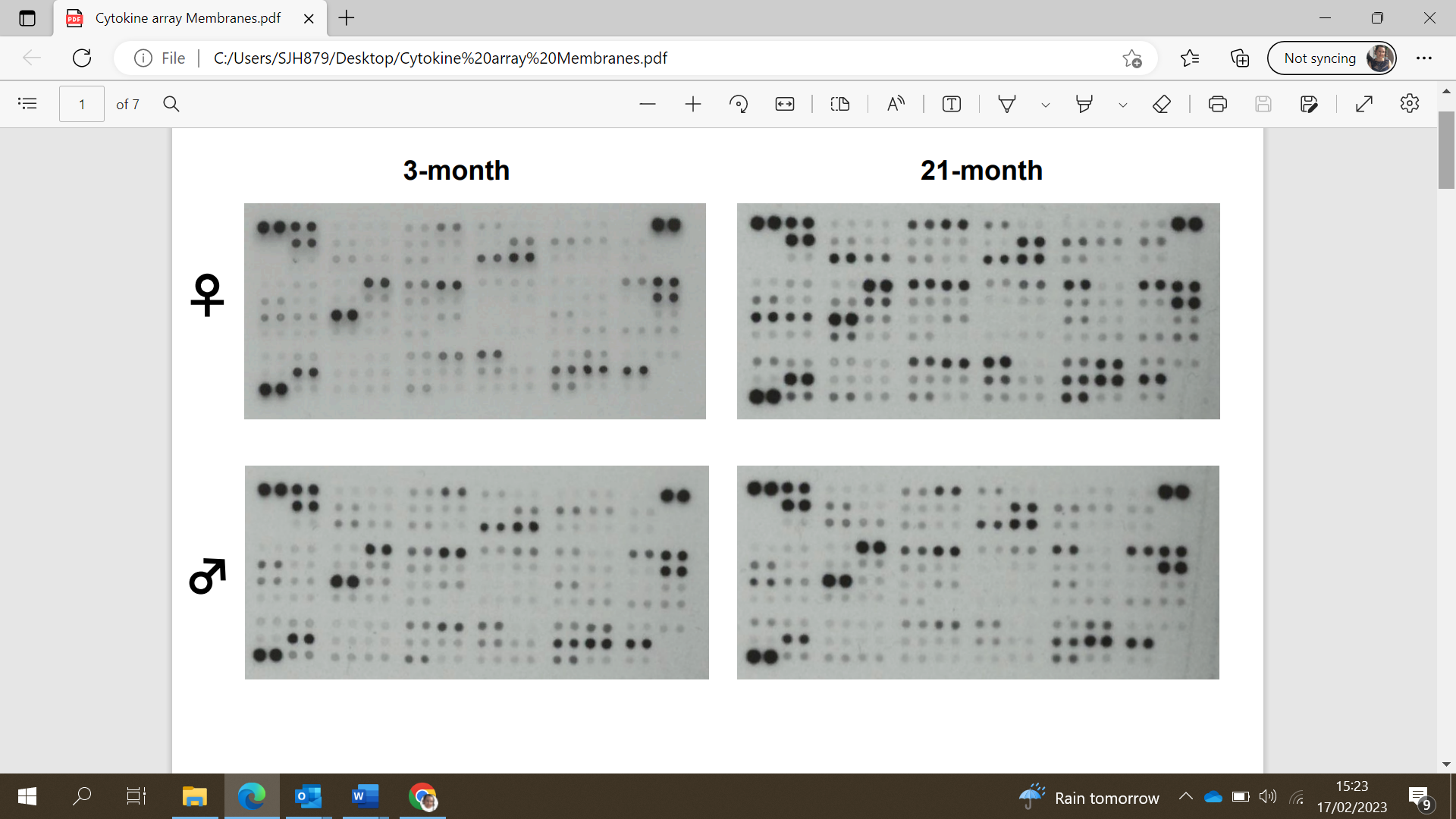


**Supplementary Figure 3**


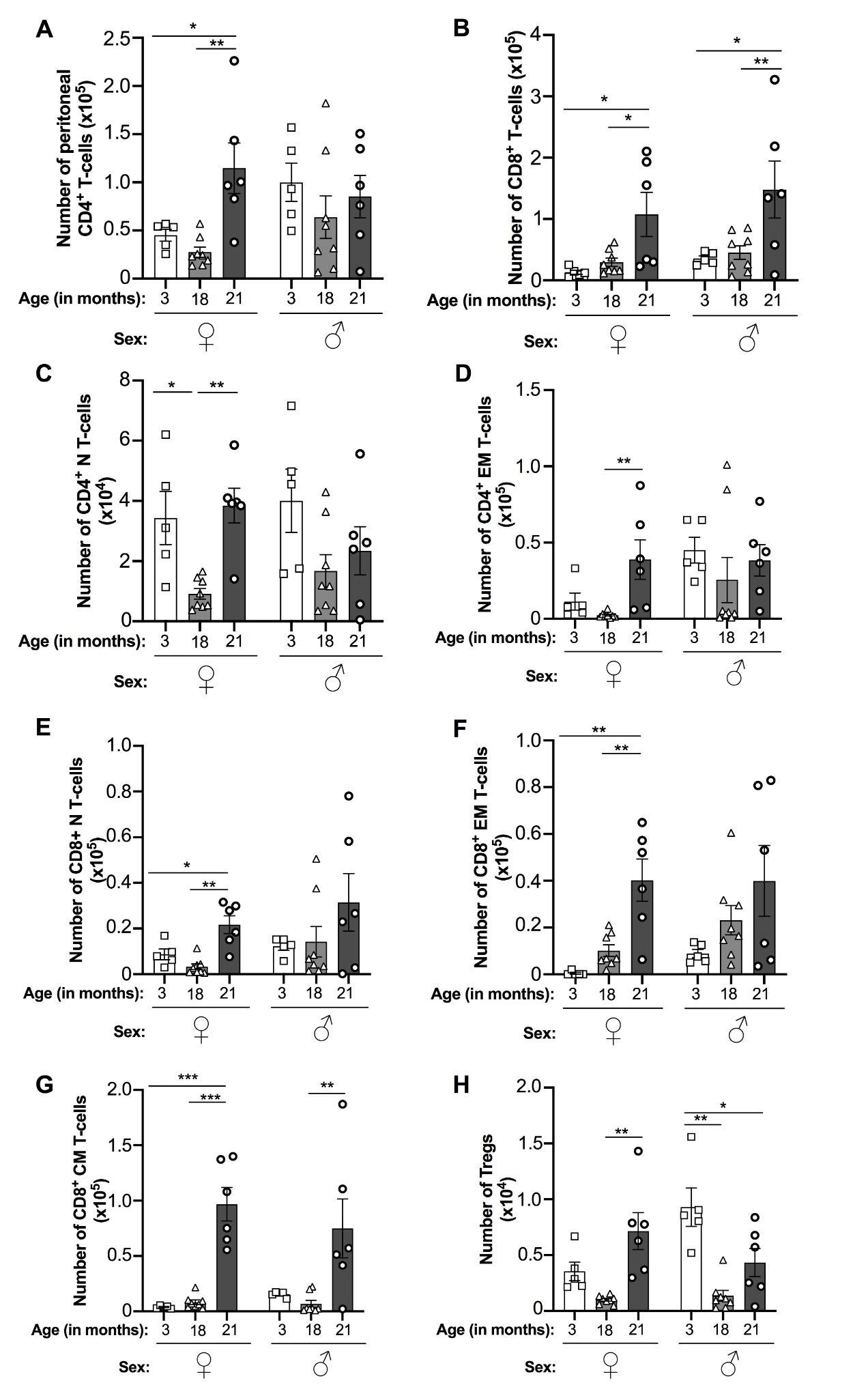


**Supplementary Figure 4**

**
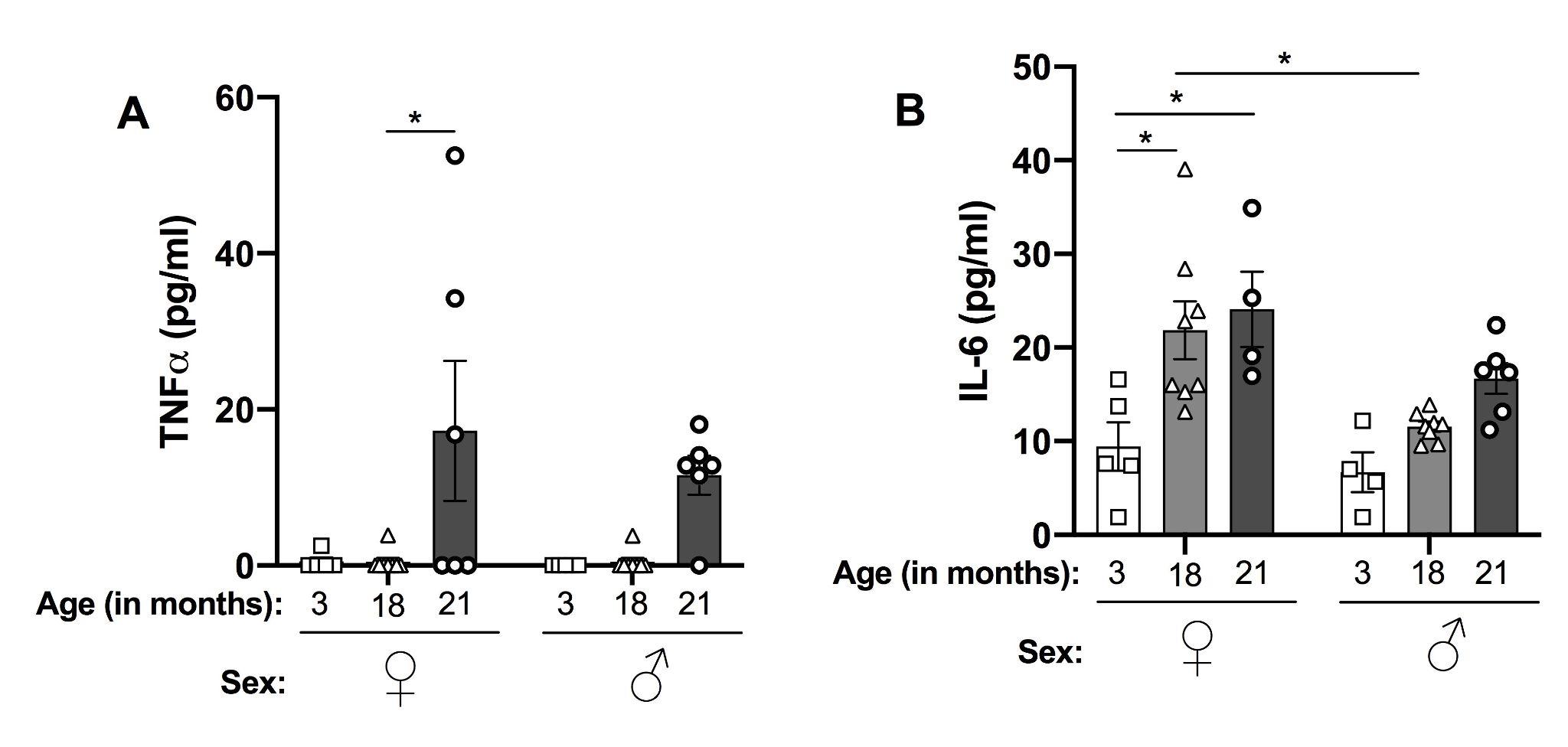
**

**Supplementary Figure 5**

**
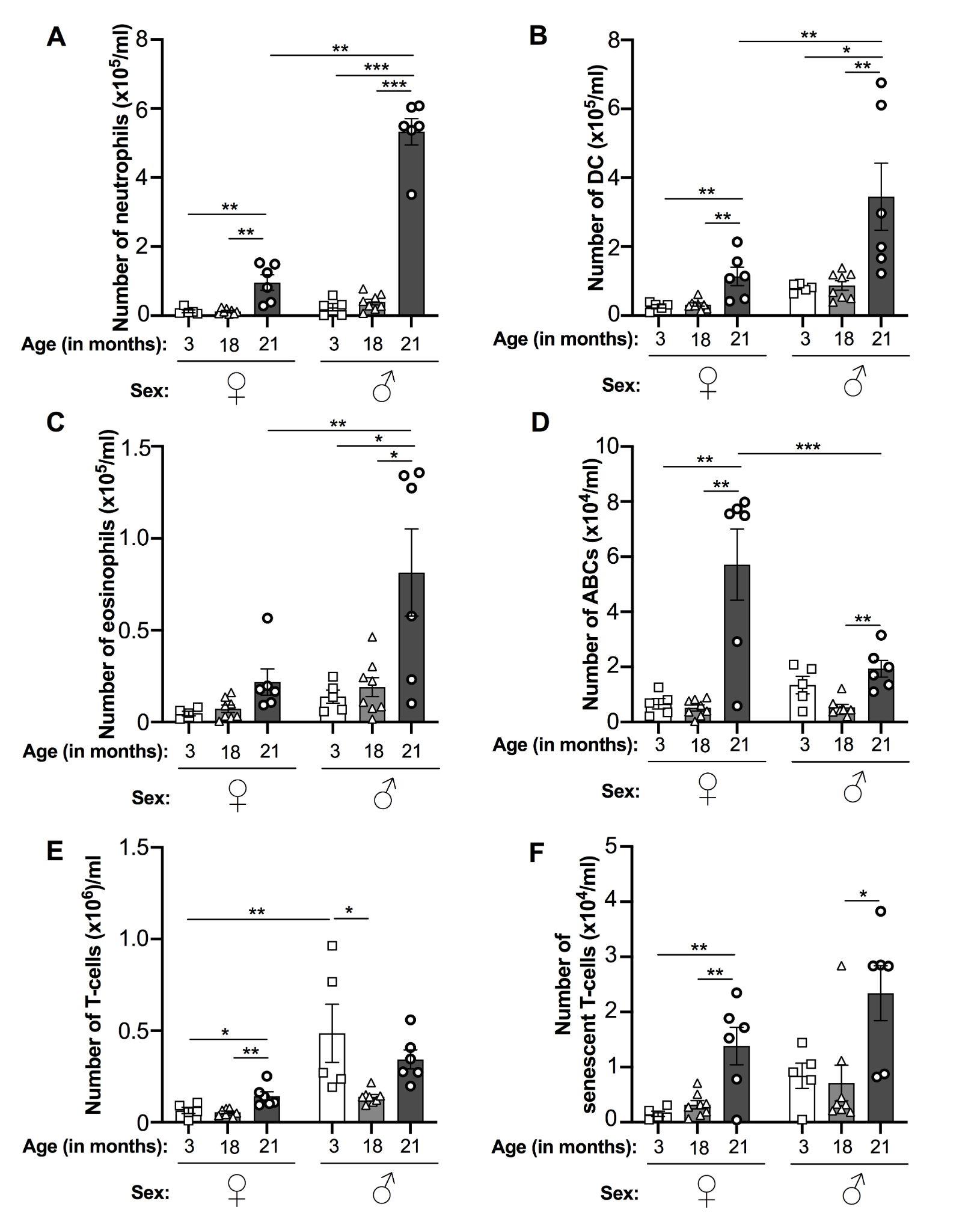
**

**Supplementary Figure 6**

**
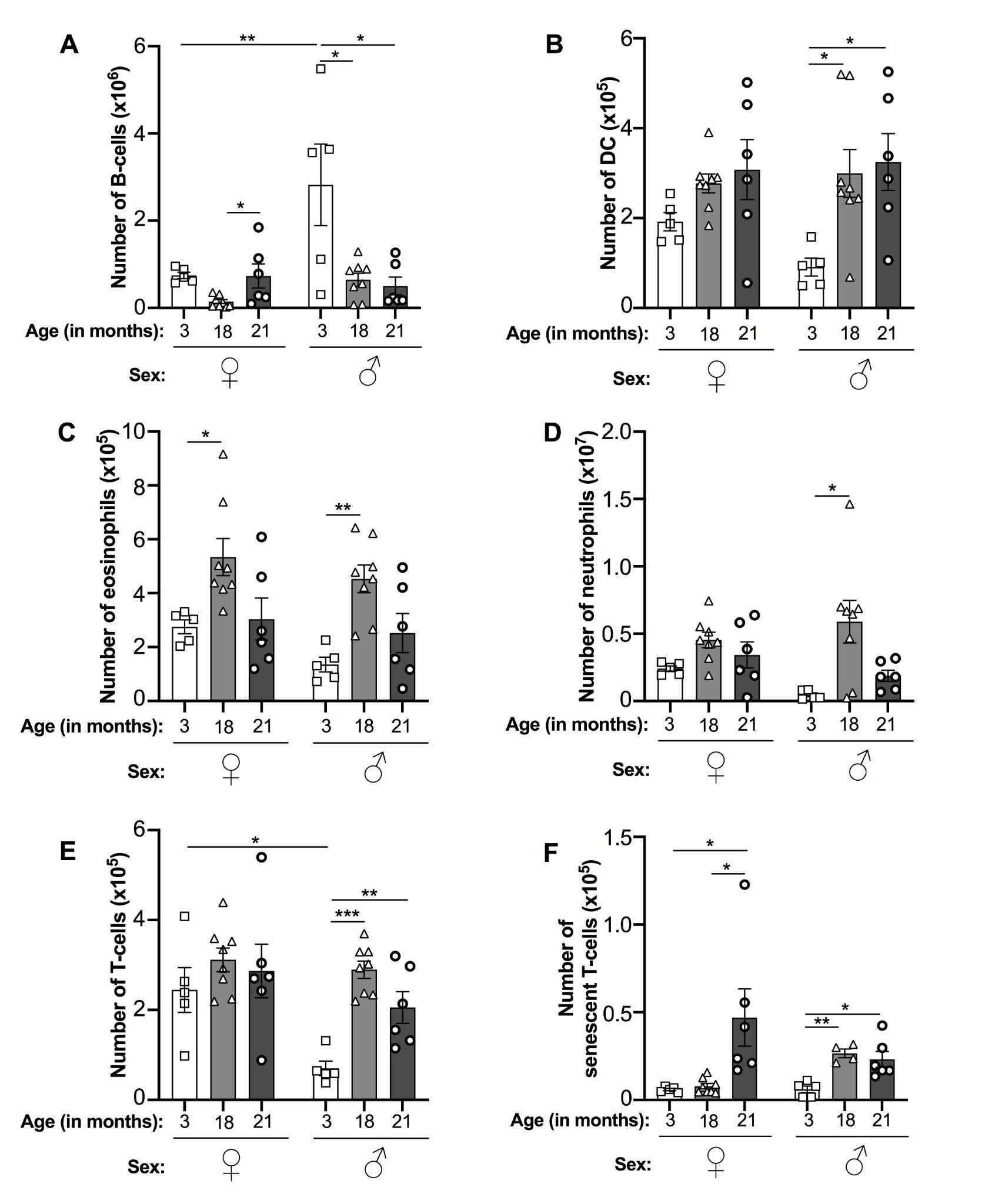
**

**Supplementary Figure 7**

**
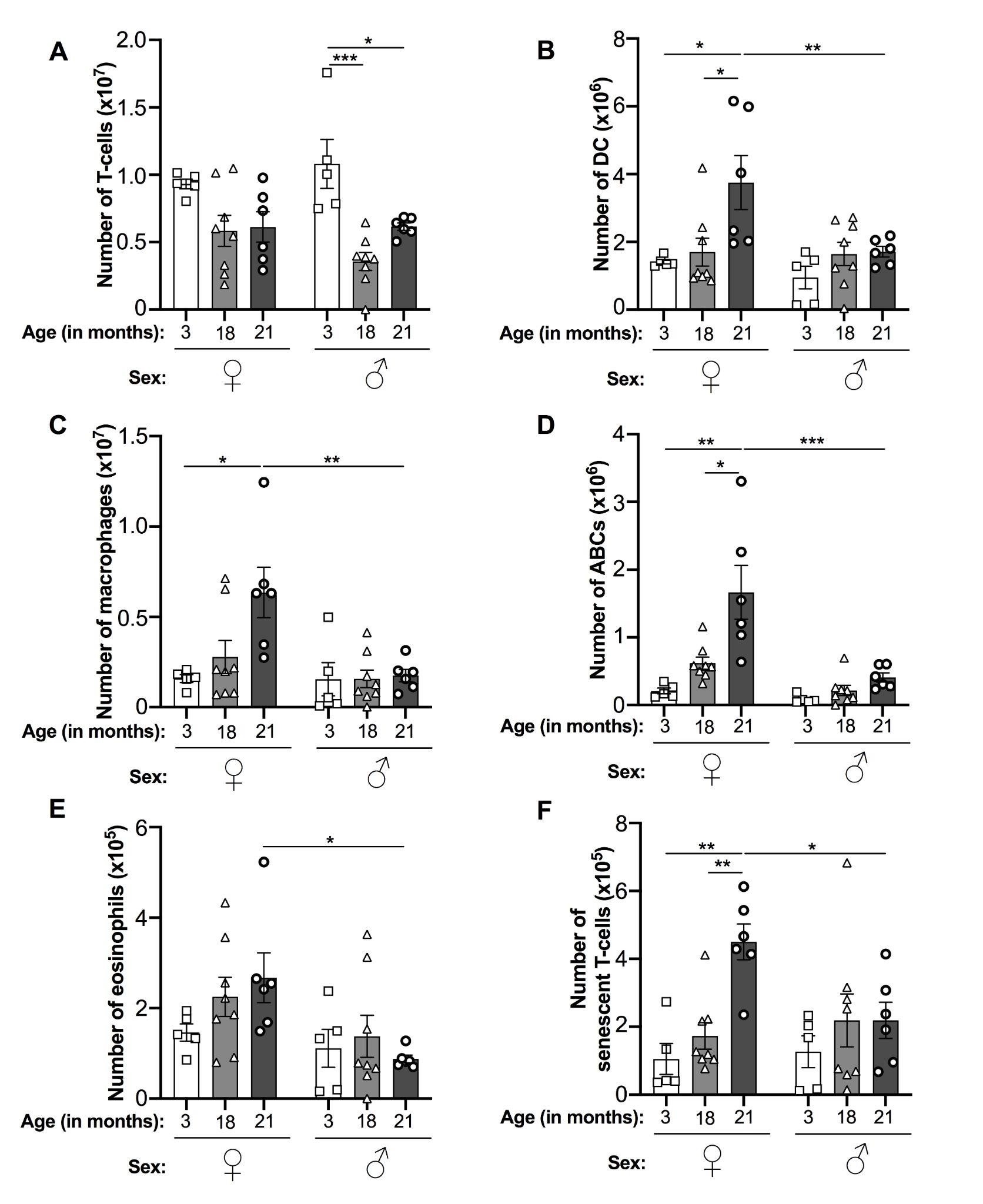
**

**Supplementary Figure 8**

**
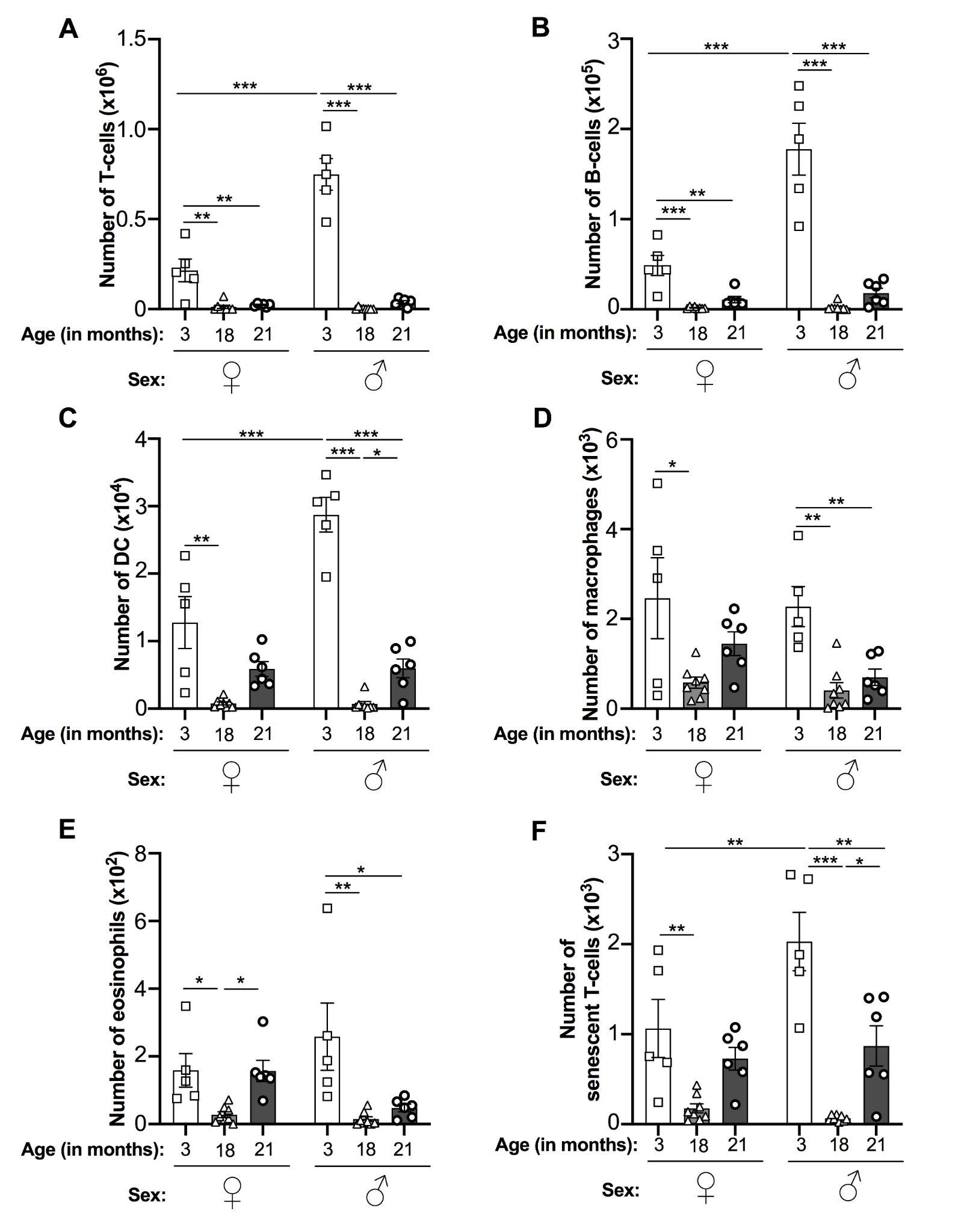
**

**Supplementary Figure 9**


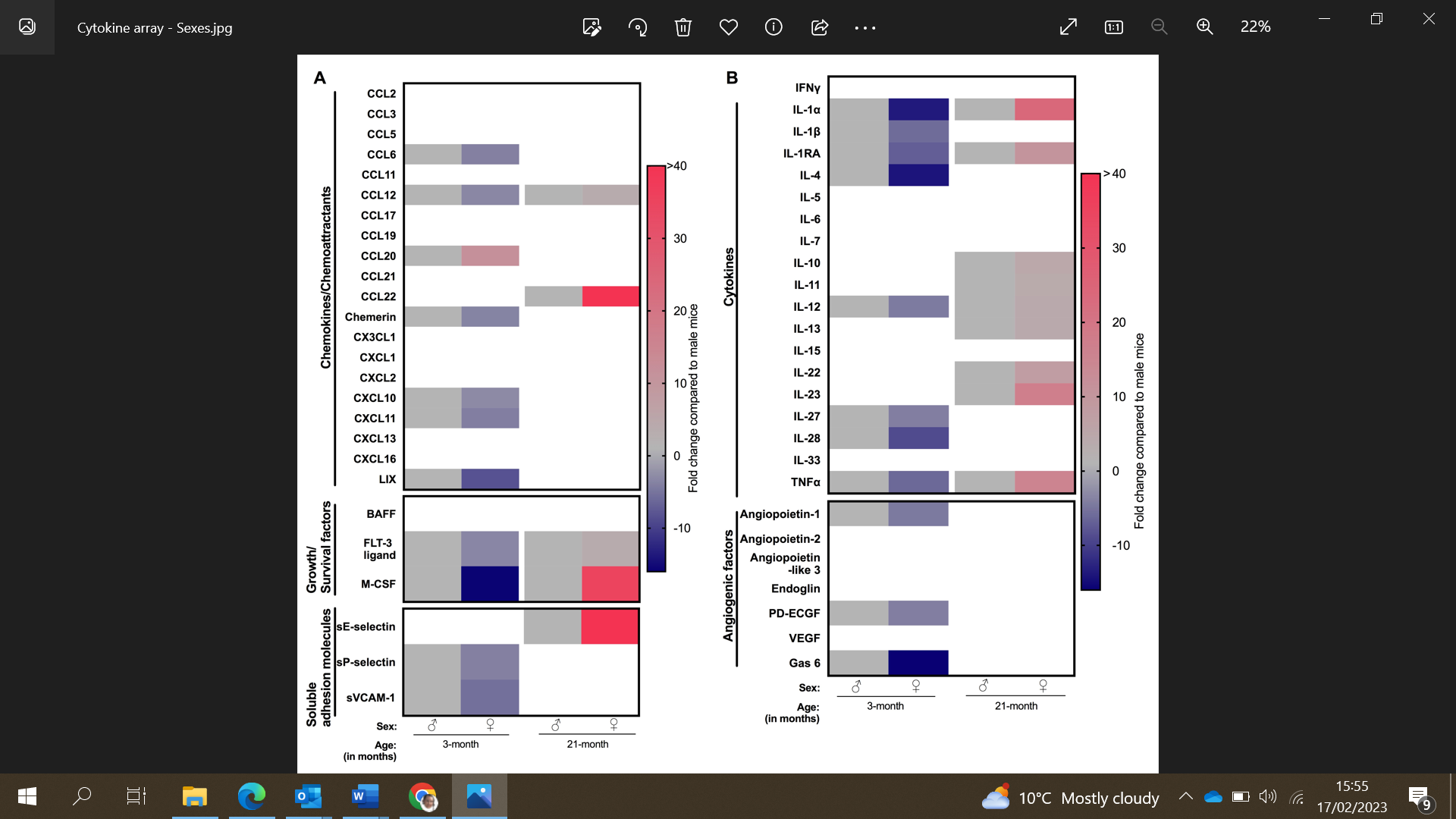


**Supplementary Figure 10**

**
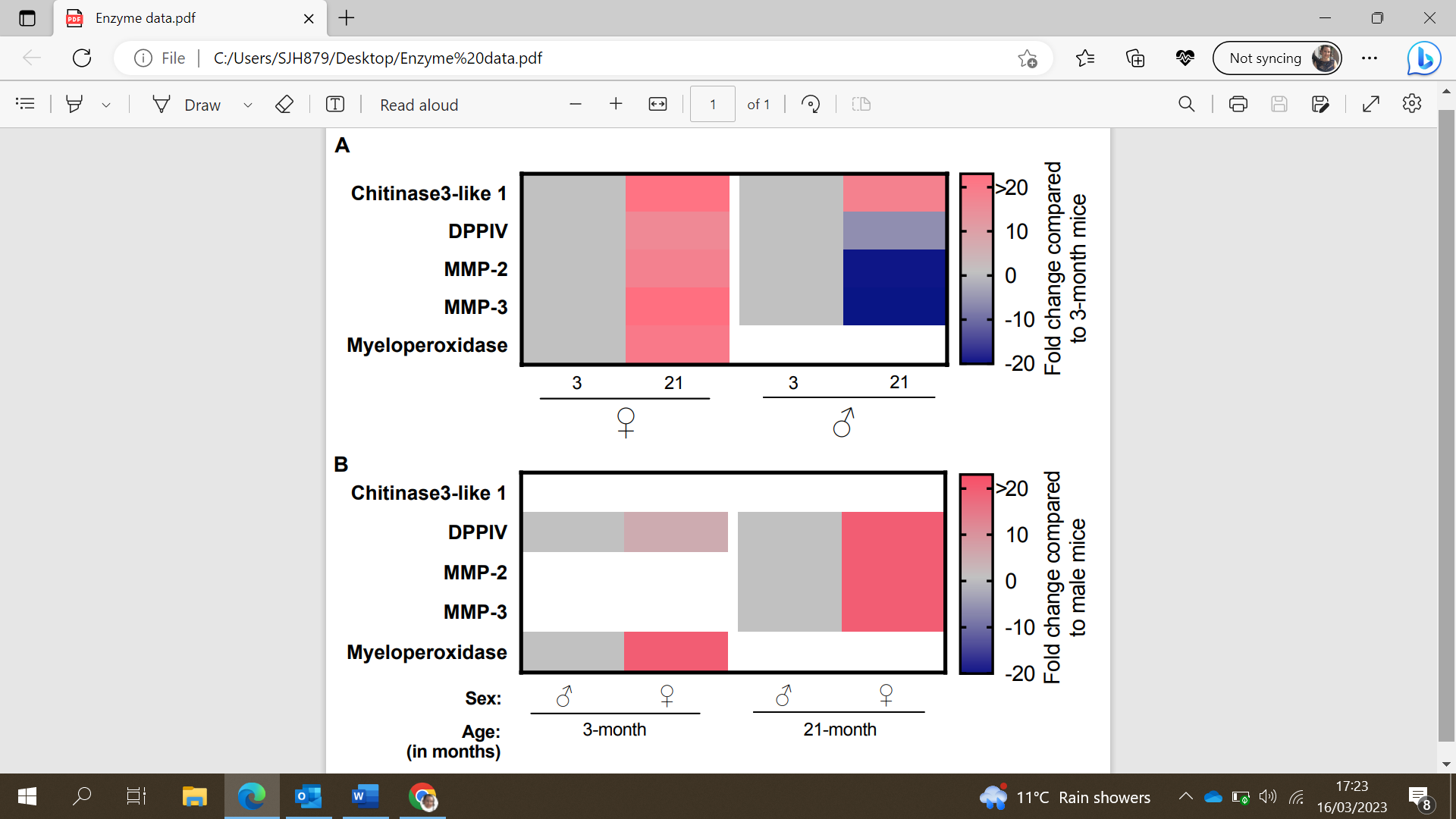
**

**Supplementary Table 1**

**
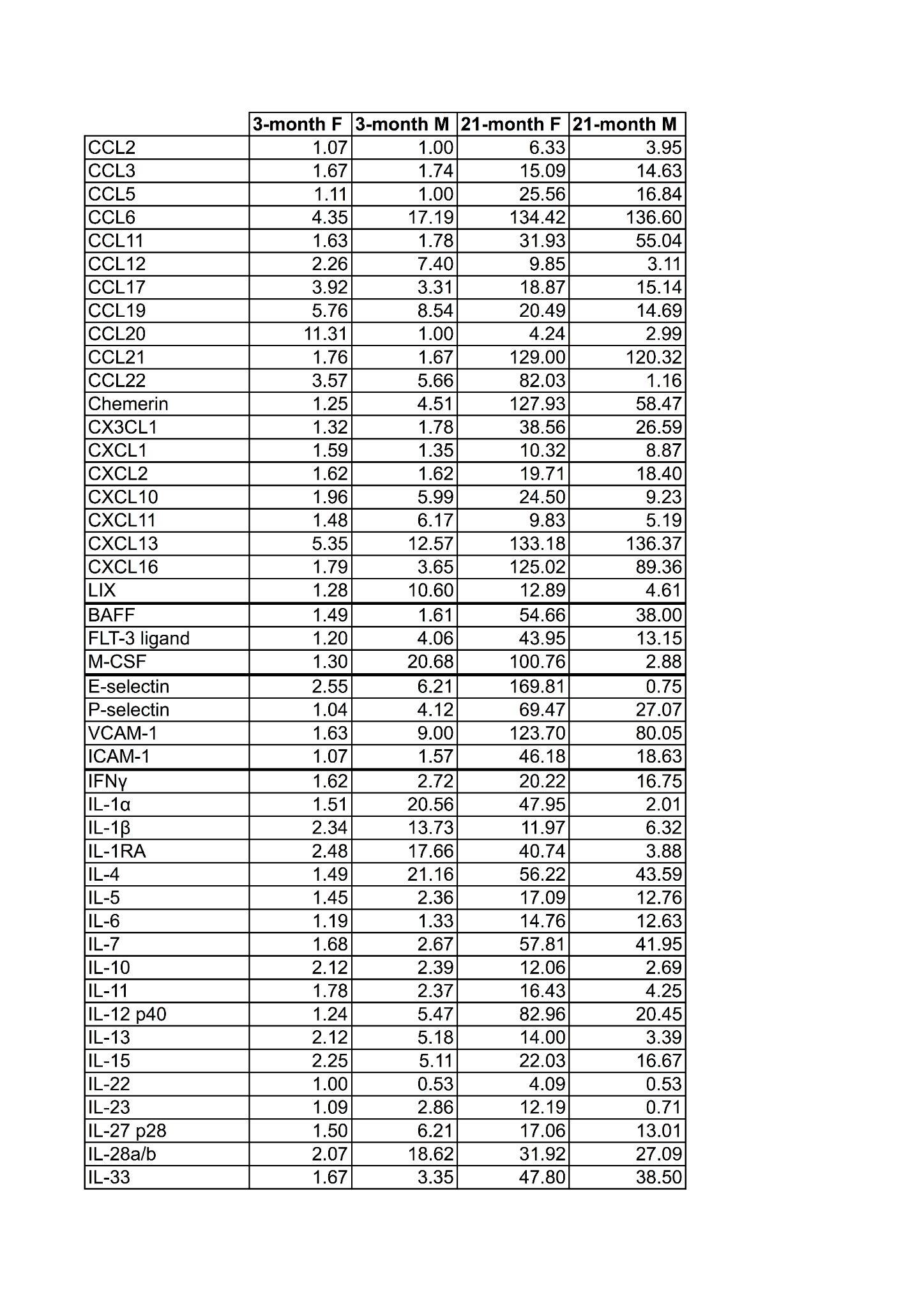
**

**Supplementary Table 1 – Cytokine array intensities of analytes**

Abundance of pro-inflammatory mediators in the peritoneal fluid of naïve 3- and 21-month female (♀) and male (♂) C57Bl6 mice were analysed using a cytokine array. Fluids from 3 mice per age group for each sex were pooled, where n=1. The cytokine membranes were analysed using ImageJ. As each analyte was present in duplicate, intensity values were extracted for each analyte spot and averaged. The background signal was removed by subtracting the average intensity value of the negative control spots from each analyte intensity value. The resultant intensity values are shown.
